# Sweet’s syndrome and mucosal prolapse polyps in a male patient with ulcerative colitis

**DOI:** 10.1186/s12876-021-02052-5

**Published:** 2021-12-16

**Authors:** Xixian Zhao, Si Jiang, Yanan Chen, Jialong Liu, Jing Liu, Xianyan Shi

**Affiliations:** 1 Department of Gastroenterology, Zhongnan Hospital of Wuhan University, No. 169, Donghu Road, Wuchang District, Wuhan, 430071 Hubei Province China; 2 Hubei Clinical Center and Key Lab of Intestinal and Colorectal Diseases, Wuhan, 430071 China; 3 Department of Dermatology and Venereology, Zhongnan Hospital of Wuhan University, Wuhan, 430071 China

**Keywords:** Classical Sweet’s syndrome, Ulcerative colitis, Mucosal prolapse polyps, Corticosteroids

## Abstract

**Background:**

Sweet’s syndrome (SS), also known as acute febrile neutrophilic dermatosis, is a rare neutrophilic dermatitis characterized by pyrexia, neutrophilia and painful papulonodular lesions with a neutrophilic dermal infiltrate.

**Case presentation:**

We presented a case report of classical SS associated with ulcerative colitis (UC) and mucosal prolapse polyps (MPPs) in a male patient.

**Conclusions:**

The particularity of this case is the occurrence of MPPs in a male patient with UC and classical SS. We also discussed whether this patient with concurrent Epstein–Barr virus infection could be treated with corticosteroids.

## Background

Ulcerative colitis (UC) is an idiopathic, chronic inflammatory disorder characterized by relapsing and remitting mucosal inflammation in colorectal mucosa [1, 2]. Diagnosis of UC is based on clinical symptoms, endoscopic and pathological features. The main symptoms of UC include bloody diarrhea, rectal bleeding, urgency, tenesmus, fecal incontinence, abdominal pain and fever [3]. Almost 50% of UC patients will develop extraintestinal manifestations during their lifetime [4]. Skin manifestations, such as pyoderma gangrenosum, bowel-associated dermatosis-arthritis syndrome, aseptic abscess ulcers, pyodermatitis–pyostomatitis vegetans and Sweet’s syndrome (SS), could be found in UC [5–8]. SS, as a rare skin manifestation, needs to be diagnosed promptly to avoid life-threatening conditions [9]. We reported the first case of a male with classical SS associated with UC and mucosal prolapse polyps (MPPs).

## Case presentation

**Fig. 1:**
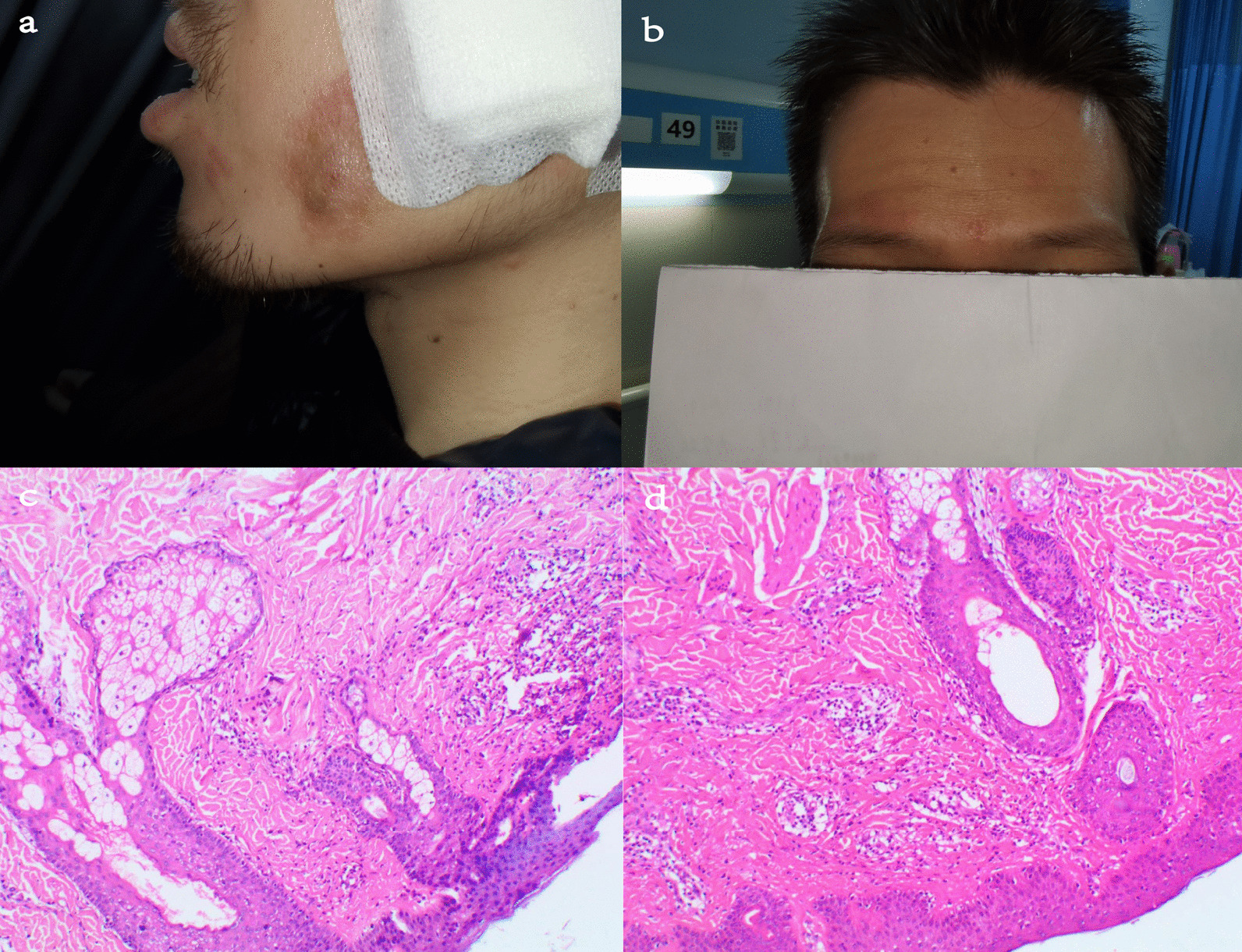
**a**, **b** Sweet's syndrome skin lesions in this patient with classical Sweet's syndrome. **c**, **d** Histologic findings for the skin lesions showing localized epidermal ulceration with neutrophil infiltration and dermal appendages with the infiltration of chronic inflammatory cells and neutrophils (H&E stain)

**Fig. 2:**
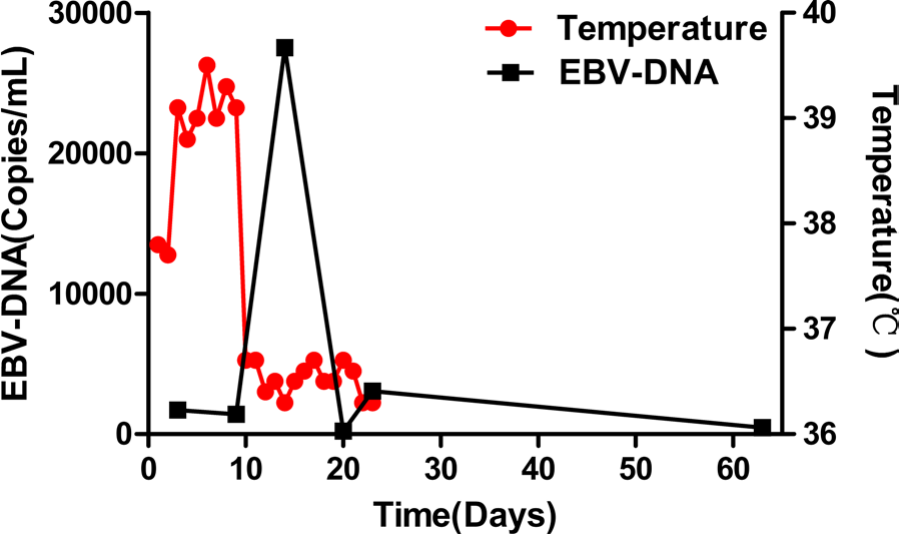
Levels of Epstein–Barr virus DNA in blood and body temperatures after admission to our hospital

**Fig. 3:**
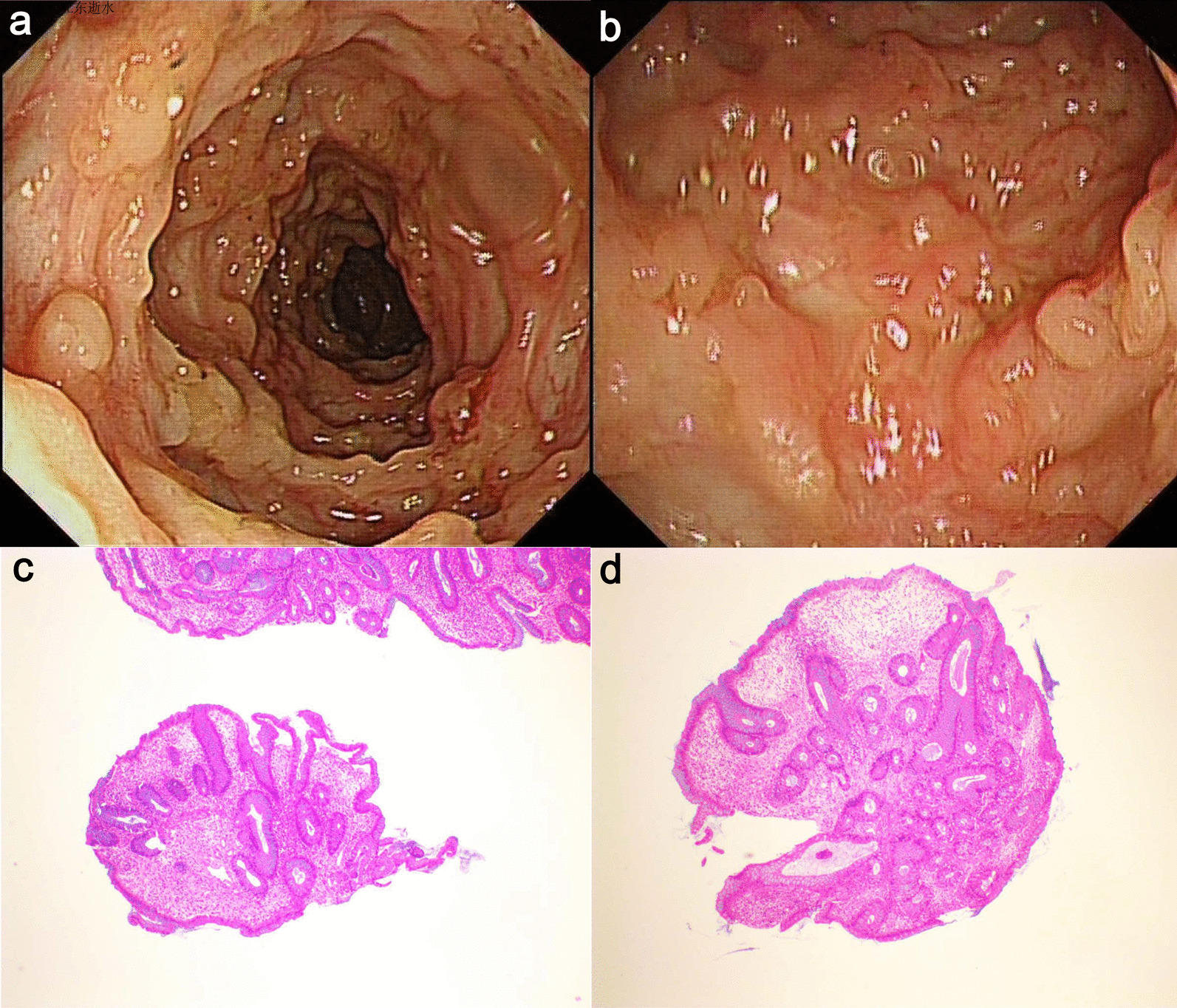
**a**, **b** Colonoscopic findings for the mucosal prolapse polyps showing scatter polypoid hyperplasia from the ascending colon to the sigmoid colon. **c**, **d** Histologic findings for the mucosal prolapse polyps showing crypt dilatation, branching, twisting with interstitial edema, local interstitial fibrosis, muscle fiber penetration growth, and localised neutrophils infiltrated into the epithelium to form cryptonitis (H&E stain)

A 31-year-old man with UC was presented to a local hospital due to diarrhea and hematochezia. The young man was diagnosed with UC 3 years ago but didn’t receive regular treatment as prescribed. After the treatment of mesalazine, anti-infection medicines of amoxicillin, parenteral nutrition supplementation and protecting the intestinal mucosa, all the symptoms worsened, and the patient began to develop fever and facial erythema with blisters forming at the raised border of the erythema (Fig. 1a, b). For further treatment, the patient was referred to our hospital with the complaints of bloody purulent stool for 1 month, fever for 9 days, erythema and blisters on face for 7 days. The initial laboratory examination demonstrated an elevated white blood cell count (10.60 × 10^9^/µL, normal range 3.5–9.5 × 10^9^/µL), increased C-reactive protein (173.96 mg/L, normal range 0–10 mg/L), procalcitonin (1.93 ng/mL, normal range < 0.05 ng/mL) and Epstein-Barr virus (EBV) DNA (1720 copies/mL, normal range 0 copies/mL) levels, together with a low haemoglobin (90.00 g/L, normal range 130–175 g/L) level. The computed tomography with contrast medium exhibited extensive colonic wall thickening with a few perienteral exudative changes and multiple lymph nodes in the retroperitoneal and mesangial areas, consistent with the characteristics of UC. Computed Tomographic Enterography showed extensive colonic thickening wall with a few perienteric exudative inflammation, and multiple lymph nodes in retroperitoneal and mesangial areas, which were consistent with the characteristics of UC. Biopsies of cutaneous lesions were performed, revealing localized epidermal ulceration with neutrophil infiltration and dermal appendages with the infiltration of chronic inflammatory cells and neutrophils (Fig. 1c, d). We considered the clinical diagnosis of acute febrile neutrophilic dermatosis. After ruling out other infectious diseases and lymphoproliferative syndrome, we decided to initiate corticosteroid treatment though high levels of EBV DNA. After that, the patient no longer developed fever and the skin manifestations improved significantly (Fig. 2). Colonoscopy revealed scatter polypoid hyperplasia from the ascending colon to the sigmoid colon (Fig. 3a, b). Histology of the resected polyp was characterized by crypt dilatation, branching, twisting with interstitial edema, local interstitial fibrosis, and muscle fiber penetration growth. And localised neutrophils infiltrated into the epithelium to form cryptonitis. These histological results were consistent with the characteristics of MPPs (Fig. 3c, d). No recurrence of SS occurred within 3 months.

## Discussion and conclusion

Classical SS, which is a subtype of SS, can be idiopathic and associated with inflammatory bowel diseases (IBDs), infections or drug intake [10]. SS may be associated with Crohn’s disease [11], and in our report, a case of classical SS associated with UC was presented. The patient was diagnosed with Classic SS based on the criteria recommended in the guidelines, including two major criteria: (1) Abrupt onset of painful erythematous plaques or nodules; and (2) Histopathologic evidence of a dense neutrophilic infiltrate without evidence of leukocytoclastic vasculitis; as well as four minor criteria: (1) Fever > 38 °C; (2) Associated with inflammatory disease or pregnancy or preceded by upper respiratory infection, gastrointestinal infection, or vaccination; (3) Excellent response to treatment with systemic glucocorticoids or potassium iodide; and (4) Abnormal laboratory values at presentation (three of four of the following): a. Erythrocyte sedimentation rate > 20 mm/h; b. Positive C-reactive protein; c. > 8000 leukocytes per microliter; and d. > 70% neutrophils [12]. If the patients with UC develop skin symptoms, the skin manifestations of UC, such as pyoderma gangrenosum, SS, bowel-associated dermatosis-arthritis syndrome, aseptic abscess ulcers, and pyodermatitis-pyostomatitis vegetans, must be excluded [13]. Moreover, it is necessary to make a skin biopsy for better diagnosis.

The first-line therapy in classical SS consists of systemic corticotherapy [10], and the second line therapeutic agents can be Cyclosporine, Dapsone, Colchicine or Indomethacin [14–17]. There was also a case in which the patient benefited from tacrolimus therapy but failed to respond to steroids [18]. Though there is evidence that corticosteroid treatment could increase the incidence of EBV infection in patients with UC, this patient needs corticosteroid to control the SS first, which might be fatal [19–21]. Although the load of EBV DNA in the blood was elevated during the administration of corticosteroid, EBV DNA finally turned negative in this case due to its self-limiting feature (Fig. 2).

Some cases of SS in UC have been reported in the literatures. However, there were few reports of colonic MPPs in patients with SS and UC. SS in UC may be associated with flare-ups of UC in most cases, but sometimes occurred in inactive UC [22–24]. Skin lesions of SS are believed to share common pathogenic mechanisms with the underlying intestinal disease in IBDs. MPPs are rare inflammatory lesions which are part of the mucosal prolapse syndrome [25, 26]. MPPs were considered to be associated with UC [27]. So far, no malignant transformation of MPPs has been reported, and no consensus has been reached on the standard treatment of MPPs as well. The clinical importance of MPPs lies in the fact that they may lead to recurrence, intestinal hemorrhage or obstructive symptoms, in which hence surgical resection is a reasonable option. Since this patient did not have such symptoms at present, we chose regular follow-up monitoring. This patient requires regular colonoscopy to monitor UC and MPPs.

This is a rare case of SS associated with UC and MPPs in a young male. We emphasized the importance of skin biopsy in dermatological manifestations of UC. Systemic corticosteroids are an effective treatment for SS associated with UC though the elevated EBV DNA load in the blood. Colonoscopy should be performed regularly to monitor the UC and MPPs.

## Data Availability

All data generated or analysed during this study are included in this published article.

## Abbreviations

SS: Sweet’ syndrome
MPPs: Mucosal prolapse polyps
UC: Ulcerative colitis
IBD: Inflammatory bowel disease
EBV: Epstein–Barr virus
